# Correction: Mikhailova, E.O. Green Silver Nanoparticles: An Antibacterial Mechanism. *Antibiotics* 2025, *14*, 5

**DOI:** 10.3390/antibiotics14111092

**Published:** 2025-10-30

**Authors:** Ekaterina O. Mikhailova

**Affiliations:** Institute of Innovation Management, Kazan National Research Technological University, K. Marx Street 68, 420015 Kazan, Russia; katyushka.glukhova@gmail.com

**Error in Figure**

In the original publication [1], there was a mistake in Figure 4. Due to an author’s technical error during the preparation, the figure showed mitochondrial dysfunction, which should be excluded since mitochondria are absent in bacteria. The production of ATP in bacteria was corrected in the figure. The corrected Figure 4 is shown below.

**Text Correction**

There was an error in the original publication [1]. The unnecessary word “vacuoles” should be deleted. 

A correction has been made to the first paragraph of Section 3. The corrected sentence is as follows: 

“…(3) penetration inside the cell and damage of intracellular structures (ribosomes); …”

The authors state that the scientific conclusions are unaffected. This correction was approved by the Academic Editor. The original publication has also been updated.

## Figures and Tables

**Figure 4:**
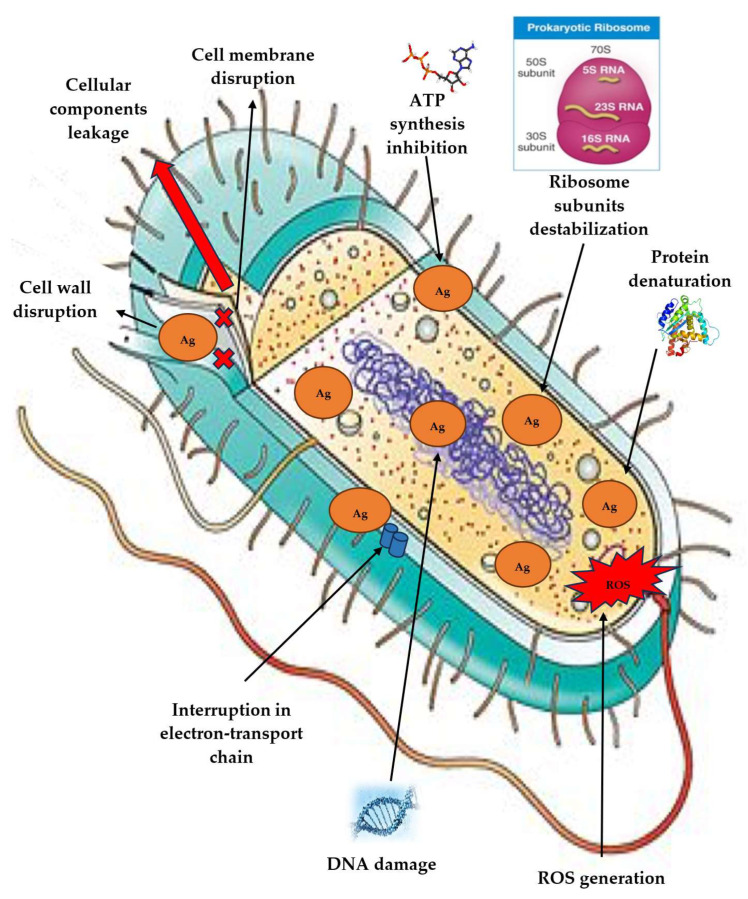
The proposal mechanism of AgNP antibacterial activity.
